# Progress towards Achieving the Recommendations of the Commission on Ending Childhood Obesity: A Comprehensive Review and Analysis of Current Policies, Actions and Implementation Gaps in Thailand

**DOI:** 10.3390/nu13061927

**Published:** 2021-06-03

**Authors:** Sirinya Phulkerd, Parichat Nakraksa, Ladda Mo-suwan, Mark Lawrence

**Affiliations:** 1 Institute for Population and Social Research, Mahidol University, Phutthamonthon, Nakhon Pathom 73170, Thailand; pbpaioegy@hotmail.com; 2 Department of Pediatrics, Faculty of Medicine, Prince of Songkla University, Hat Yai, Songkhla 90110, Thailand; laddamosuwan@gmail.com; 3 Institute for Physical Activity and Nutrition (IPAN), School of Exercise and Nutrition Sciences, Deakin University, Geelong 3220, Australia; mark.lawrence@deakin.edu.au

**Keywords:** childhood obesity, government policy, Thailand

## Abstract

Despite a significant commitment to tackling childhood overweight and obesity, questions remain about the progress the Thai Government has made in implementing childhood obesity prevention policies and actions. This study aimed to review and assess the implementation of the government’s policies and actions for childhood obesity prevention in Thailand compared with the recommendations of the Commission on Ending Childhood Obesity and to identify the implementation gaps. Policy data were collected from governmental and NGO websites and publications and via direct contact with government officials. Stakeholder meetings were held to seek further information and advice on implementation gaps and to give recommendations. The analysis of each policy was conducted against pre-determined criteria formulated from literature assessments and stakeholder consultations. The policies and actions that were implemented by the Government were consistent with 33 broad policy actions and 55 specific policy actions. Preconception and pregnancy care was the policy area that was most implemented. Six broad policy actions were assessed as ‘high’ performance, these were: sugar-sweetened beverage taxation, nutrient labeling, nutrition guidance for preconception and pregnancy care, the International Code of Marketing of Breast-milk Substitutes, regulatory measures for supporting maternal breastfeeding, and regulations on the marketing of complementary foods and beverages. Policy coherence and monitoring and evaluation (M&E) were identified as major implementation gaps. Increasing the effectiveness of childhood obesity prevention in Thailand will require national immediate attention towards building infrastructure to enhance coherence among the policies and to put in place M&E mechanisms for each policy.

## 1. Introduction

Childhood obesity has risen dramatically over the last few decades, in both high-income and low-to-middle income countries. Almost half of children under five years old with overweight or obesity are Asian [1]. In 2014, the Director-General of the World Health Organization (WHO) established the Commission on Ending Childhood Obesity (ECHO) to address this challenge. Over the following years, ECHO developed a comprehensive, integrated package of recommendations and an implementation plan [2,3] to tackle childhood obesity. ECHO synthesized recommendations from a variety of existing global obesity prevention reports and focused on a whole-of-government and life course approach to obesity prevention [4]. To date, a few developed countries have reported on their implementation of national policies and actions consistent with ECHO [5,6].

The prevalence of overweight in under-5s in Thailand has reduced from 10.9% in 2012 to 8.2% in 2016 [7]. This trend indicates Thailand is meeting the global nutrition target of preventing an increase in the prevalence of overweight among under-5s. This achievement is at least partly due to the Thai Universal Health Coverage (UHC) policy that was first implemented in 2002 [8]. Maternal and child health issues are part of a benefit package under UHC that aims to improve the quality of life of mother and child. However, challenges still remain in older children. The prevalence of overweight and obesity has increased 2.4-fold among children aged 6–14 years between 1996 and 2014 [9,10,11,12]. In recognition of the contribution of obesity to premature death and disability in adulthood, a number of national actions based on ECHO recommendations were developed to help promote child and youth health, including preventing overweight and obesity. Governments, health and non-health authorities in collaboration with non-governmental organizations (NGOs), which are organizations that operate independently of government, developed a range of actions to tackle childhood obesity. These actions included the Miracle of 1000 Days Policy developed by the Department of Health (under the Ministry of Public Health) in collaboration with the Ministry of Social Development and Human Security, the Ministry of Education, and the Ministry of Interior [13], a policy to ban soda sales in schools developed by the Department of Health in collaboration with the Ministry of Education, the Sweet Enough Network of Thailand and the Thai Health Promotion Foundation [14], and sugar-sweetened beverage taxation by the Ministry of Finance in collaboration with the Ministry of Public Health, the Sweet Enough Network and their academic and NGO partners [14].

In 2016, the Royal Thai Government adopted the ‘20-Year National Strategy (2017–2037)’ that includes actions to promote life-cycle development from early life through health and relational environments [15]. In the following year, the Government adopted the 12th National Economic and Social Development Plan (2017–2021) that set goals and indicators to achieve the objectives and targets of the National Strategy. Among the Strategy’s indicators for strengthening and realizing the potential of human capital is the reduction of overweight and obesity among Thai people [16]. The Thai Department of Health also developed a set of indicators for monitoring the nutritional status of Thai people which includes the assessment of childhood overweight and obesity in schoolchildren aged 0–5 years and 6–14 years [17]. 

In 2018, the Department of Health launched the Five-year National Nutrition Action Plan (2018–2022) mainly in response to Sustainable Development Goals (SDGs), Global Nutrition Targets and Non-communicable disease (NCD) Global Action Plan [18]. The plan sets a number of goals and expected outcomes by achieving SDG2.2 (end all forms of malnutrition by 2030) and SDG3.4 (reduce by one-third pre-mature mortality from NCDs through prevention and treatment and promote mental health and wellbeing by 2030), Global Nutrition Targets 1, 4 and 5 (reduction in stunting, no increase in childhood overweight and increase in the rate of exclusive breastfeeding, respectively), and NCD voluntary global target 7 (halting the rise in diabetes and obesity) [18].

In the same year, the Department of Health also launched the 2018–2030 National Physical Activity Plan, the first national plan to promote sufficient physical activity in the Thai population in order to address obesity and related NCDs in Thailand by creating built environments and support systems [19]. The plan sets a number of goals and indicators that need to be accomplished by 2030, such as 95% of young children (aged 0–5 years) having normal gross motor development, 40% of children and adolescents (aged 6–17 years) having sufficient physical activity, 80% of adults and elderly (aged 18 years and above) having sufficient physical activity, and people (aged 6 years and above) having not more than 13 h of daily total sedentary behavior. To enhance implementation of the plan, country- and community-wide campaigns were planned and have been implemented and supported by the Thai Health Promotion Foundation and its partners. The campaigns promote physical activity by combining a variety of strategies, such as media coverage and promotions, education, community and school events, and policy and programmatic initiatives [20].

Despite its significant commitment to tackling childhood overweight and obesity, questions remain about the progress the Thai Government has made in implementing childhood obesity prevention policies and policy actions based on the ECHO framework, and the key challenges it faces in implementing the policies. This paper aims to: (1) review current childhood obesity prevention policies and actions by Thai governments; (2) assess these government policies and actions compared to ECHO recommendations; and (3) identify policy implementation gaps in Thailand.

## 2. Materials and Methods

### 2.1. Data Collection and Verification

#### Document Assessment

This step was adapted from the approach developed by an INFORMAS Food-EPI protocol for assessing government policies and actions related to obesity and non-communicable disease prevention conducted in Thailand [21]. Data for national-level implementation were gathered for six areas of policy actions recommended by ECHO: (1) Promote intake of healthy foods; (2) Promote physical activity; (3) Preconception and pregnancy care; (4) Early childhood diet and physical activity; (5) Health, nutrition and physical activity for school-age children; and (6) Weight management of children with obesity [3]. They consist of 36 broad policy actions with 67 specific policy actions, in total. 

Relevant policy documents were collected by submitting official information requests, searching websites and publications (including official codified policies) of governmental and non-governmental organizations, searching Thai newspapers, and via direct contact with government officials. Policy data that were collected in this study were defined as the current government policies in place from June 2019 (the date at which evidence collection for the project started) to March 2020 (the date at which the evidence collection finished). We were able to measure whether implementation of policy activities was occurring because this information was recorded by the government agency and available as part of the process of collecting data on relevant policy documents.

After gathering all the data, a verification meeting was conducted with government officials to verify the completeness and accuracy of the policy data. Fourteen representatives from 14 Bureaus/Divisions/Offices at the Ministries of Public Health, Education, Interior, Finance, Social Development and Human Security, and Labor, and the Office of the Prime Minister were officially invited to the meeting. After the meeting, the research team followed up with some stakeholders for additional information. The team also arranged another in-person meeting with the missed stakeholders. 

### 2.2. Data Analysis

A content analysis was applied to describe the details of each policy action compared with ECHO recommendations and to identify implementation gaps of each policy action using the pre-defined criteria. This study selected criteria for analyzing policy implementation gaps in Thailand based on literature and consultation with a Thai stakeholder group (described in the following section). Five key factors that were evident as especially influential factors for success or failure of policy implementation were selected as criteria for analysis. These five criteria were: policy comprehensiveness [22,23]; coverage [24,25]; monitoring and evaluation mechanism [14,26]; multi-sectoral collaboration [14,25,27]; and coherence [14,28,29,30]. Overall, the analysis of each policy or action resulted in their categorization into one or other of three levels: high, H (having at least three criteria rated as high level and no criteria as low level); moderate, M (having at least two criteria rated as high level); low, L (having less than two criteria rated as high level) according to a consensus among stakeholders. The description of the criterion and the basis of the performance rating is set out in Table 1.

### 2.3. Preparation of Recommendations

A Thai Stakeholder Advisory Committee was formed to advise and make recommendations to the research team, including sharing their expertise and serving as conduits of information to and from their organizations and networks. The Committee consisted of three senior government officials, four university professors, one representative of public organizations, and two leaders of NGOs, who have direct experience in public health and childhood obesity-related policy making, implementation and/or monitoring and evaluation in Thailand. 

The stakeholder meetings were organized to seek advice and further information during the processes to support the verifying of completeness and accuracy of the policy evidence gathered, the implementation of performance criteria drafting, the analysis of policy implementation gaps, and the finalizing of study results.

**Figure 1:**
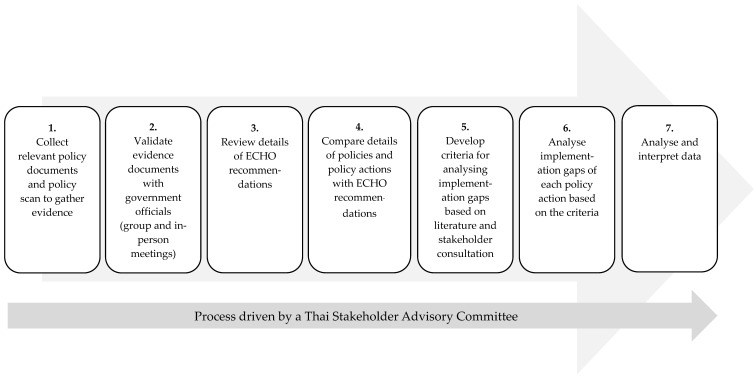
The seven-step process for data collection and analysis.

Figure 1 presents the taken steps for reviewing and analyzing the existing policies and actions and implementation gaps in Thailand.

## 3. Results

### 3.1. Government Policy Implementation against ECHO Recommendations

**Figure 2:**
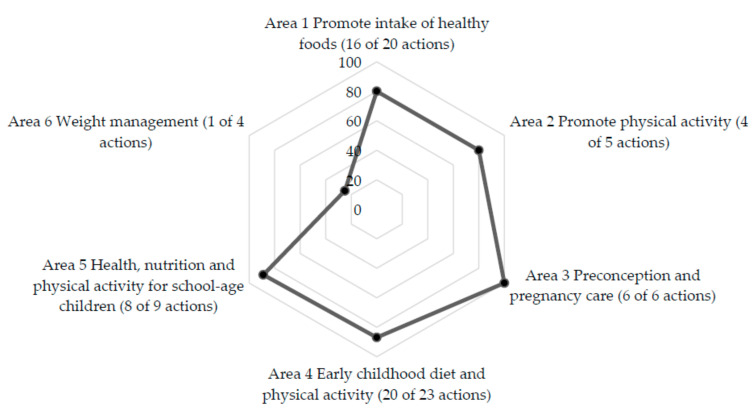
Specific policy actions implemented in Thailand in each of the policy areas.

Table 2 summarizes the presence or absence and overall assessment of the 36 broad policy actions and 67 specific policy actions organized under six policy areas of ECHO recommendations. The policies and actions that were implemented by the Thai Government were consistent with 33 broad policy actions and 55 specific policy actions. Figure 2 illustrates a proportion of specific policy actions that were implemented in Thailand. 

Three broad policy actions from the set of recommendations on the marketing of foods and non-alcoholic beverages to children (1.3), the impact of cross-border marketing of unhealthy foods and beverages (1.5), and food education (4.10) were not in place. In specific policy actions, twelve actions (1.3.1–1.3.3, 1.5.1, 2.1.4, 4.5.1, 4.10.1–4.10.2, 5.4.1, 6.1.2–6.1.4) were not implemented in Thailand. A description of the policy implementation in each area is presented in Supplementary Table S1.

Among the six areas, Area 3, ‘Preconception and pregnancy care,’ was the most implemented area. All recommended, broad and specific policy actions in this area took place in Thailand. One specific policy action on nutrition in guidance and advice (3.3) was a score H.

Both Area 2, ‘Promote physical activity,’ and Area 5, ‘Promote health, nutrition and physical activity for school-age children,’ lacked one specific action each: Peer education and whole-of-school initiatives (2.1.4); and Development of nutrition, food and health education curricula (5.4.1), respectively. Two specific policy actions in Area 2 were given a score H, which are an effective tax on sugar-sweetened beverages and a standardized global nutrient-labeling system. No specific policy action in these two areas was assessed as H.

Many specific policy actions were absent in Area 1, ‘Promote intake of healthy food,’ specifically, actions aligned with 1.3.1–1.3.3 ‘Implementation of the set of recommendations on the marketing of foods and non-alcoholic beverages to children’; and 1.5.1 ‘Engage in intercountry discussions on policies and proposals for regulating cross-border marketing of unhealthy foods and non-alcoholic beverages to children’. Two specific policy actions were given a score of H, which are an effective tax on sugar-sweetened beverages (1.2.2) and a standardized global nutrient-labeling system (1.6.2). 

For Area 4, ‘Promote early childhood diet and physical activity,’ all specific policy actions were absent for: 4.5.1 ‘Assess the impact of legislation, regulations and guidelines to address the marketing of complementary foods for infants and young children’; 4.10.1 ‘Develop nutrition, food and health education curricula’; and 4.10.2 ‘Integrate nutrition and health education components into the core curriculum’. Three specific policy actions in this area were rated as H, which are regulatory measures for the International Code of Marketing of Breast-milk Substitutes (4.1), regulatory measures for supporting maternal breastfeeding (4.4.1), and regulations on the marketing of complementary foods and beverages (4.5.2 and 4.5.3).

For Area 6, ‘Weight management of children with obesity,’ all specific policy actions were absent for: 6.1.2 ‘Align services with existing clinical guidelines’; 6.1.3 ‘Educate and train concerned primary health care providers’; and 6.1.4 ‘Include childhood weight management services as part of universal health coverage’. No specific policy action in this area had a score of H.

### 3.2. Identification of Policy Implementation Gaps

**Figure 3:**
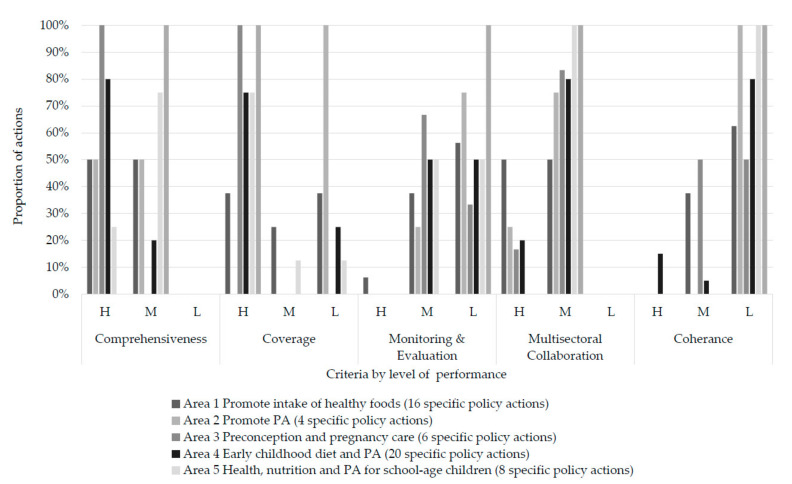
Assessment of policy areas and actions implemented in Thailand by the five criteria for analyzing policy implementation gaps (H—high, M—moderate, L—low).

Figure 3 illustrates the assessment of the government performance in implementing each specific policy action that was categorized into three levels using five criteria. Overall, 24 specific policy actions (of 55 specific policy actions) were assessed with ‘low’ performance; 14 were assessed as ‘moderate’; and seven were assessed as ‘high’.

Across the five criteria, policy coherence and monitoring and evaluation were identified as major implementation gaps. At least 50% of the currently specific policy actions in each policy area were assessed as ‘low’ performance for policy coherence. Similarly, monitoring and evaluation was assessed as being low in at least 50% of the specific policy actions in each policy area, except Area 3. The only specific policy actions that were assessed with ‘high’ performance for these criteria were actions in Area 4 (4.4.1 and 4.5.2–4.5.3) for policy coherence and in Area 1 (1.8.1) for monitoring and evaluation (Table S1). Another gap identified in this study was policy coverage. Of particular concern was that all specific policy actions in Area 2 were assessed as ‘low’ coverage. 

All the specific policy actions performed well for comprehensiveness and multisectoral collaboration. They were assessed as ‘high’ or ‘moderate’ for these two criteria. Many specific policy actions were implemented in accordance with ECHO recommendations. At least 50% of the specific policy actions in each area were assessed as having ‘high’ comprehensiveness, except Area 5 (24% of its specific policy actions). At least 50% of the specific policy actions in each area were assessed as ‘moderate’ for multisectoral collaboration. 

## 4. Discussion

This study reviewed and analyzed the implementation of available government policies and actions for childhood obesity prevention in Thailand compared with the framework of ECHO recommendations. Overall, the implementation of Thai governments covered the majority of the recommended components in each policy area. Despite a good performance, it is not likely sufficient yet to halt the rise of childhood obesity. Infrastructure support was identified as a major implementation gap, in particular a lack of policy coherence and policy M&E mechanism. 

Thailand has implemented many policy actions relating to ECHO recommendations. This may be due to various national strategies and plans taking place in Thailand. The national strategies and plans provide a comprehensive framework for actions in a congruous drive to achieve an intended goal. As such, responsible government agencies are mandated to develop actions and align their activities with the national strategies and plans. This is consistent with results from other countries, such as Canada and Scotland which had food and/or nutrition plans that had more policies relating to ECHO recommendations [5]. Thailand has more downstream (individual-level behavioral approach) and midstream (organization-level approach) policy actions than upstream (affecting large populations) policies, similar to other countries such as Australia [5]. Key challenges in moving upstream or addressing upstream factors in policies may include: a lack of understanding and awareness of issues; linking up policy actions across different sectors and across different levels of government; a whole-of-government approach which creates a coherence mechanism. These important factors can contribute to developing an upstream approach requiring multisectoral and intersectoral action.

The Thai Government’s policy performance was best for the policy area ‘Pre-conception and pregnancy care’. All the recommended broad and specific actions in this area were being implemented in Thailand. This performance can be explained by the impact of the implementation of the Thai UHC policy. The UHC focused on building health infrastructure and expansion of health insurance coverage that includes maternal and child health services [8]. The UHC benefit package provides maternal and child health services from the pre-conception period until children are 5 years old. Every child will have a regular check-up from health personnel at 2, 4, 6, 9, 18, 30, and 42 months of age at a well-child clinic and, for pregnant women with free access to a continuum of care including antenatal care, delivery, and postpartum care, at public hospitals [31]. It was evident that the UHC has resulted in a fairly equitable distribution of maternal and child health services [32]. This may ultimately result in a decrease in the prevalence of obesity among the under-fives, which puts Thailand on-course to meet the Global Nutrition target [7].

In common with many countries, Thailand reported no action on the restriction of marketing unhealthy foods and non-alcoholic beverages to children. The evidence suggests arguments for and against regulations to restrict this marketing by the advocacy groups—health and consumer and industries influence government decision-making. The key points of the debate concern quality and sufficiency of evidence which underpins any regulatory initiative, the range of media to be regulated, and the type of foods that are advertised and potentially subject to regulation [33]. Despite the presence of self-regulation of unhealthy food marketing to children by food industries or the Thai-pledge in 2010 [34], a failure of the Thai-pledge was reported. Among food products sold, 16.3% and 10% of sugar-sweetened beverages and snacks, respectively, were still advertised targeting children in free and digital television programs [35]. Thailand also failed to address the complex challenges of cross-border marketing (inflowing and outflowing), in part due to opposition of private sectors and a weak self-regulatory scheme [36]. This cross-border action strongly requires cooperation and harmonization of the cross-country regulation, in particular at a regional level. This will help to avoid weakening national restrictions and will strengthen government efforts to address food marketing, especially digital media, which, nowadays, is a part of all children’s worlds. 

The results of this study suggest the implementation of specific policies did not necessarily result in better outcomes if governments lack appropriate infrastructure in systems to support the implementation of policy. Even though there is alignment between the government implementation and the ECHO framework, it is still insufficient to push towards reducing childhood obesity in Thailand, as seen from the rising prevalence of childhood overweight and obesity (aged 6–14 years) since 1996 in Thailand [9,10,11,12]. Major contributors to policy implementation that were identified were a lack of policy coherence and a robust M&E system. This finding is consistent with previous evidence, indicating that a lack of capacity to pursue policy coherence is one of the main obstacles at country-level in reducing NCDs and their risk factors that a health policy alone cannot solve [37,38,39]. Therefore, government support for enhancing policy coherence across areas that impact the governance, prevention, management and surveillance of childhood obesity is essentially part of effective implementation. A platform for interaction to create opportunities to advance the dialogue on policy coherence on solid ground is needed. The health sector can act as a key advocate for enhancing policy coherence within and between policy actors that have a bearing on the prevention of childhood obesity. 

The lack of a robust M&E system is another key challenge for policy implementation in Thailand. Measuring government performance, tracking its progress and evaluating strategies and policies towards achieving intended goals are critical to ensure effective implementation [40]. Despite the presence of Thai population censuses and national surveys on health and its determinants, this surveillance system alone is unlikely to be sufficient to track government progress or to enhance the effectiveness of implementation, especially where the policy is complex, multisectoral and long-term. A better understanding is needed of the process of which policy moves and how. The most basic requirement for establishing a robust M&E system is a demand for the system [40]. Therefore, it is important for Thailand to gain interest from stakeholders and their commitment for such a system to be established and take hold in the country. This needs implementation support through strong political will and institutional capacity to accelerate progress. Capacity in the workforce, in particular, is needed to develop, support and sustain the M&E system. Technical assistance and training for capacity and institutional development may be required.

The ECHO framework provided an opportunity for tackling childhood obesity in each country, taking into account the life-course approach, prevention and treatment and obesogenic environments to improve population health and health equity [4]. Although the framework was developed based on other reports, and specifically recommended priority actions that are the potential leverage to accelerate the implementation, this may limit it for addressing other related issues. For example, the framework is more likely to focus on single nutrient interventions rather than improving dietary patterns although its beneficial effect on obesity is well evident [41,42]. Despite the importance of governance, the framework does not sufficiently reflect in action, in particular support planning and investment in backbone infrastructure, such as institutional and workforce capacities, inter-ministerial cooperation and coordination, M&E systems, accountability and transparency, and avoidance of conflicts of interest that are a basic foundation to accelerate progress. Moreover, the framework was designed specifically for a planning stage of the policy cycle that does not aim to measure levels of implementation and assess the progress that the government has made over time. This study suggests a need for complementing the framework with other appropriate measures to assess other dimensions of childhood obesity and the level of policy implementation. Currently, there are several assessment tools developed, such as HEPA PAT [43], Food-EPI tool [44] and BIA-Obesity tool [45], that could be used to assess public-sector or private-sector policies and commitments at national and sub-national levels, with appropriate tailoring of assessment measures to the country context. 

This study has some limitations. Firstly, the study obtained data based on publicly available information and was supplemented with stakeholder interview. Some unrecorded information relied only on stakeholder recall and thus may account for recall bias and have missed some information. This may, therefore, affect the assessment results of policies and actions. Secondly, the study may have missed other policies and actions that are beyond the ECHO recommendations, such as district-level policies that have formulated policies through less formal approaches and may not be publicly accessible and thus were not captured in the study. Lastly, this study did not aim to provide an in-depth understanding of how and why the policies have or have not been successfully developed, although that is of importance for policy learning and improvement. However, this study provided a platform for interaction between policymakers and stakeholders that allow them to better understand government performance and the actors across sectors. It also helped the stakeholders to determine where to focus to fill in implementation gaps.

## 5. Conclusions

An important step in promoting the prevention and control of a country’s childhood obesity is to understand the government’s performance and progress against policy areas, broad and specific policy actions, and to identify implementation gaps. This study found that the Thai Government’s implementation of policy activities is in line with the majority of ECHO recommendations. However, it is likely still not sufficient to halt the rise of childhood obesity according to national evidence showing a continuous rise in the prevalence of childhood overweight and obesity (aged 6–14 years). The study identified the lack of policy coherence and crucial infrastructure aspects, in particular, policy M&E systems as possibly slowing progress. Therefore, instituting policy coherence is essentially important. The Government needs to raise awareness among stakeholders about policy coherence and discuss emerging approaches on institutional mechanisms to enable integrated policy-making for tackling childhood obesity, as well as putting in place robust M&E systems. A core requirement will be good governance including government commitment, institutional and workforce capacities, accountability and transparency, and resources. Further research is needed to assess public-sector or private-sector policies and commitments, at national and sub-national levels, using other assessment tools.

## Figures and Tables

**Table 1:** Description of each criterion and their performance rating for analyzing policy implementation gaps.

Criteria	Description of Each Criterion	Rating Scale (Performance Level and Definition)
1. Policy comprehensiveness	Refers to elements of ECHO that are contained in the policy content.	- high = containing all recommended elements (and beyond the recommendations, H+) - moderate = containing some of the recommended elements - low = containing no recommended element or no data available
2. Policy coverage	Refers to a level that a policy/action can reach target group/setting.	- high = 80–100% of target group/setting - moderate = 50–79% of target group/setting - low = less than 50% of target group/setting
3. Monitoring and evaluation mechanism (M&E)	Refers to the availability of monitoring and process and outcome evaluation plan in each policy/action.	- high = formal M&E mechanism/platform available for measuring both process and outcome indicators in the policy - moderate = formal M&E mechanism/platform available for measuring process or outcome indicators in the policy - low = no M&E plan
4. Multi-sectoral collaboration	Refers to the level of collaboration between organizations in different areas of policy (e.g., health, social, environment) and different sectors (e.g., public, private, third), as well as communities and people, working together to achieve policy outcomes.	- high = collaboration between organizations in all different areas of policy, different sectors and different levels such as central and local - moderate = collaboration between organizations in some different areas of policy, sectors, or levels - low = no collaboration
5. Policy coherence	Refers to the definition of OECD [29] which is the creation of a systematic promotion of mutually reinforcing policy actions across government departments and agencies creating synergies towards achieving the agreed objectives.	- high = institutional mechanism available to support across all stages of the policy coherence building block - moderate = institutional mechanism available to support some stage(s) of the policy coherence building block - low = occasional meeting or no mechanism

**Table 2:** Policy area, broad and specific policy actions of ECHO recommendations—their implementation in Thailand and their overall assessment.

ECHO Recommendations			Thailand's Implementation Based on ECHO Recommendations	Overall Assessment of Thailand Implementation
Policy Area	Broad Policy Action	Specific Policy Action		
Area 1 Promote intake of healthy foods	1.1 Appropriate and context-specific nutrition information and guidelines	1.1.1	✓	L
		1.1.2	✓	L
		1.1.3	✓	M
		1.1.4	✓	L
	1.2 An effective tax on sugar-sweetened beverages.	1.2.1	✓	M
		1.2.2	✓	H
	1.3 The set of recommendations on the marketing of foods and non-alcoholic beverages to children	1.3.1	NA	NA
		1.3.2	NA	NA
		1.3.3	NA	NA
	1.4 Nutrient profiles to identify unhealthy foods and beverages	1.4.1	✓	L
	1.5 Impact of cross-border marketing of unhealthy foods and beverages	1.5.1	NA	NA
	1.6 A standardized global nutrient-labeling system	1.6.1	✓	M
		1.6.2	✓	H
	1.7 Interpretive front-of-pack labeling	1.7.1	✓	M
		1.7.2	✓	M
	1.8 Healthy food environments in child settings	1.8.1	✓	M
		1.8.2	✓	M
	1.9 Access to healthy foods in disadvantaged communities	1.9.1	✓	L
		1.9.2	✓	L
		1.9.3	✓	L
Area 2 Promote physical activity	2.1 Guidance to children and adolescents, their parents, carers, teachers and health professionals	2.1.1	✓	L
		2.1.2	✓	L
		2.1.3	✓	L
		2.1.4	NA	NA
	2.2 Facilities on school premises and in public spaces for physical activity during recreational time	2.2.1	✓	M
Area 3 Preconception and pregnancy care	3.1 Diagnosis and management of hyperglycemia and gestational hypertension	3.1.1	✓	M
	3.2 Monitoring and management of gestational weight gain	3.2.1	✓	M
	3.3 Appropriate nutrition in guidance and advice	-	✓	H
	3.4 Guidance and support for the promotion of good nutrition, healthy diets and physical activity	3.4.1	✓	M
		3.4.2	✓	M
		3.4.3	✓	M
Area 4 Early childhood diet and physical activity	4.1 Regulatory measures such as the International Code of Marketing of Breast-milk Substitutes	-	✓	H
	4.2 A full practice for the Ten Steps to Successful Breastfeeding	-	✓	M
	4.3 Promotion of the benefits of breastfeeding for both mother and child	-	✓	L
	4.4 Regulatory measures for supporting maternal breastfeeding	4.4.1	✓	H
	4.5 Regulations on the marketing of complementary foods and beverages	4.5.1	NA	NA
		4.5.2	✓	H
		4.5.3	✓	H
	4.6 Guidance and support to carers to avoid specific categories of foods	-	✓	M
	4.7 Guidance and support to caregivers to encourage the consumption of a wide variety of healthy foods	-	✓	M
	4.8 Guidance to caregivers on appropriate nutrition, diet and portion size	4.8.1	✓	M
		4.8.2	✓	M
	4.9 Provision of healthy foods, beverages and snacks in formal child-care settings	4.9.1	✓	L
		4.9.2	✓	L
	4.10 Food education in the curriculum in formal child-care settings	4.10.1	NA	NA
		4.10.2	NA	NA
	4.11 Physical activity in the daily routine and curriculum in formal childcare settings	4.11.1	✓	L
		4.11.2	✓	L
	4.12 Guidance on appropriate sleep time, sedentary or screen-time, and physical activity or active play	4.12.1	✓	L
		4.12.2	✓	L
	4.13 Whole-of-community support for carers and child-care settings	4.13.1	✓	M
		4.13.2	✓	M
		4.13.3	✓	M
		4.13.4	✓	M
Area 5 Health, nutrition and physical activity for school-age children	5.1 Standards for meals provided in schools, or foods and beverages sold in schools	-	✓	M
	5.2 Elimination of provision or sale of unhealthy foods	5.2.1	✓	L
		5.2.2	✓	L
	5.3 Access to potable water in schools and sports facilities	5.3.1	✓	L
	5.4 Inclusion of nutrition and health education within the core curriculum of schools	5.4.1	NA	NA
		5.4.2	✓	L
	5.5 Nutrition literacy and skills of parents and carers.	-	✓	L
	5.6 Food preparation classes to children, their parents and carers	5.6.1	✓	L
	5.7 Physical education in the school curriculum	5.7.1	✓	M
Area 6 Weight management of children with obesity	6.1 Appropriate weight management services for children and adolescents with overweight or obesity	6.1.1	✓	L
		6.1.2	NA	NA
		6.1.3	NA	NA
		6.1.4	NA	NA

## Data Availability

Not applicable.

## Supplementary Materials

The following are available online at https://www.mdpi.com/article/10.3390/nu13061927/s1, Table S1: Assessment of current policies and actions in Thailand compared to ECHO recommendations and using five criteria.
