# Supplementary material for: Progress towards Achieving the Recommendations of the Commission on Ending Childhood Obesity: A Comprehensive Review and Analysis of Current Policies, Actions and Implementation Gaps in Thailand
Source: Nutrients. 2021 Jun 3;13(6):1927. doi: 10.3390/nu13061927 (PMC8230086; doi:10.3390/nu13061927)
Supplement: Supplementary file 1 [file nutrients-13-01927-s001.zip › nutrients-1197963-supplementary.pdf]

Table S1. Assessment of current policies and actions in Thailand compared to ECHO recommendations and using five criteria

ECHO's specific policy action	Thailand's implementation	Assessment results by criteria				
		COM	COV	M&E	MSC	COH
1.1.1 Inform the population about childhood overweight and obesity and consequences for health and well-being.	The Department of Health communicates information through public media such as Thairath, Siam Rath, Bangkok, Thai Business Post, frontline and website, agencies and network organizations. There are publications / public relations for those who are interested can find it on various websites.	H	L	L	M	L
1.1.2 Update, as necessary, guidance on the prevention of childhood obesity through the consumption of a healthy diet throughout the life course.	Implementation of the Bureau of Nutrition's (Department of Health) Guidelines for the prevention and control of over-nutrition in school children.[1]	M	M	L	M	L
1.1.3 Ensure that food-based dietary guidance is disseminated in an accessible manner for children, carers, school staff and health professionals.	- Development and implementation of Thai Food-based Dietary Guidelines (FBDGs) or shortly called "Practice with the nine principles of nutrition ". The quantitative part of the Thai FBDGs or food guide model was established as the "Nutrition Flag."[2]<br>- Development and implementation of Mother and Child Health Guideline for mothers and health professionals (Pink Book) [3]	H	H	L	H	L
1.1.4 Develop and implement evidence-based, public education campaigns about what constitutes a healthy diet and the need for it and for physical activity, which are appropriately funded and sustained over time.	Department of Health regularly promotes public campaign on healthy diets and physical activity such as recent nationwide campaigns on drinking milk and physical activity daily and other related activities at regional and provincial levels through Regional Health Promotion Centers and Public Health Province Offices.[4]	M	L	L	M	L
1.2.1 Analyse the administration and impact of a tax on sugar-sweetened beverages.	Excise Department, Ministry of Finance established a mechanism to assess the performance of sugar-sweetened beverage (SSB) industry and the income received after adoption of SSB taxation. Taxed SSB products were randomly sampled to check the sugar content in each product.	H	M	M	M	M

ECHO's specific policy action	Thailand's implementation	Assessment results by criteria				
		COM	COV	M&E	MSC	COH
1.2.2 Levy an effective tax on sugar-sweetened beverages according to WHO's guidance.	The Ministry of Finance issued the 2017 Excise Tax Act for taxation of sugar content in non-alcoholic beverages. It came into effect on September 16, 2017.[5]	H	H	M	H	M
1.3.1 Assess the impact of legislation, regulation and guidelines to tackle the marketing of unhealthy foods and non-alcoholic beverages to children, where required.	-	**Not available**				
1.3.2 Adopt, and implement effective measures, such as legislation or regulation, to restrict the marketing of foods and non-alcoholic beverages to children and thereby reduce the exposure of children and adolescents to such marketing.	-	**Not available**				
1.3.3 Establish mechanisms to effectively enforce implementation of legislation or regulation on the marketing of foods and non-alcoholic beverages to children.	-	**Not available**				
1.4.1 Establish a national nutrient-profiling model to regulate marketing, taxation, labelling and provision in public institutions, based on WHO's regional or global nutrient-profile models.	The Bureau of Nutrition, Department of Health in collaboration with the Institute of Nutrition, Mahidol University developed nutrient profiling system as criteria for nutrient-based classification of healthy and unhealthy foods in Thailand. This system has been used for developing food and nutrition policies, such as food labeling policy.[6]	M	L	L	M	L
1.5.1 Engage in intercountry discussions on policies and proposals for regulating cross-border marketing of unhealthy foods and non-alcoholic beverages to children through WHO regional		**Not available**				

ECHO's specific policy action	Thailand's implementation	Assessment results by criteria				
		COM	COV	M&E	MSC	COH
committees and other relevant regional mechanisms.						
1.6.1 At the international level, work through the Codex Alimentarius Commission to develop a standardized system of food labelling, to support health literacy education efforts through mandatory labelling for all pre-packaged foods and beverages.	Thai Food and Drug Administration (FDA) implemented the food labeling system in accordance with the Codex standard on front-of-pack nutrition labelling. The FDA developed the Thai criteria on nutrients - % energy and fat according to the Codex criteria, while %sugar was obtained from a meeting with Department of Health and food industry. The system was pretested before being introduced.<br><br>Codex has established a FAO / WHO Food Standards Program Commission (Codex Alimentarius Commission-CAC) located at the National Bureau of Agricultural Commodity and Food Standards. Ministry of Agriculture and Cooperatives. Thailand is a member of the Codex, with the NCPO having the duty to circulate or communicate about food standards that are international standards, while the FDA is overseeing and enacting laws related to food standards.[7]	M	H	M	H	M
1.6.2 At the domestic level, adopt mandatory laws and regulations for nutrition labelling.	Adoption of a Notification of the Ministry of Public Health to enforce the nutrition labeling according to Issue No. 182[8], 219[9] and 392[10] under the Food Act, B.E. 2522, which has penalties directed as well.	H	H	M	H	M
1.7.1 Consider undertaking pre-market/consumer testing of interpretive front-of-pack labelling, based on a nutrient-profile model.	The Thai FDA has a process in pretesting each front-of-pack labelling format that it developed before putting it into action.	H	L	M	H	M

ECHO's specific policy action	Thailand's implementation	Assessment results by criteria				
		COM	COV	M&E	MSC	COH
1.7.2 Adopt, or develop as necessary, a mandatory interpretive front-of-pack labelling system based on the best available evidence to identify the healthfulness of foods and beverages.	Adoption of a Notification of the Food and Drug Administration Re: Notification of Ministry of Public Health No. 394(11) (B.E. 2561), issued under the Food Act B.E. 2522, Re: Foods that must show the label of nutrition and energy, sugar, fat and sodium form GDA.[11]	H	M	M	H	M
1.8.1 Set standards for the foods that can be provided or sold in child-care settings, schools, children's sports facilities and at events (see also recommendations 4.9 and 5.1) based on a national nutrient-profile model.	- The National Early Childhood Development Committee developed the Food Standards for child care center, which later was approved by the Cabinet on January 2, 2019. This standard is part of the National Early Childhood Development Center Standard. The Office of the Education Council Secretariat acts as a focal point for following up implementation of the Standard.[12]<br>- Development of a school lunch management standard that was developed based on nutrition and food hygiene and safety standards for primary school in Thailand.[13]<br>- Development of a food, snacks, milk and drinks classification guide for children age 3-15 years (revised version) by Bureau of Nutrition.[14]<br>- Development of a school lunch management guideline for single dish menu, developed by Nutrition Behavior Development Division, Bureau of Nutrition.[15]<br>- Development of the Health Promotion School Standards and the Health Literacy Promotion School Standards, by Bureau of Health Promotion, Department of Health.[16]<br>- Development of recipes for children of school age, by Ministry of Education.[17]<br>- Development of Guidelines for promoting nutrition and sanitation of children and youth in remote areas (for community leaders), by Funds for Development of Children and Youth in Remote Areas, Department of Livestock Development.[18]	M	H	L	H	L

ECHO's specific policy action	Thailand's implementation	Assessment results by criteria				
		COM	COV	M&E	MSC	COH
1.8.2 Apply such food laws, regulations and standards in catering services for existing school, child-care and other relevant settings.	Development of Thai School Lunch Software Program for assisting healthy and nutritious school lunch provision for school children, by Institute of Nutrition and Thailand's National Electronics and Computer Technology Center under the National Science and Technology Development Agency, Ministry of Higher Education, Science, Research and Innovation.[13]	M	H	H	H	L
1.9.1 Involve actors and resources outside the health system to improve access, availability and affordability of nutritious foods at a sustained scale in disadvantaged communities (for instance, through incentives to retailers and zoning policies).	- Development and implementation of Blue Flag Project (Tong-Fa) by Department of Internal Trade that gives Thai people the right to access food at a low price. It is also in conjunction with a state welfare card that helps low-income people to easier access food.[19]<br>- Development and implementation of a subsidy project for raising newborn children by the Department of Children and Youth Ministry of Social Development and Human Security.[20]<br>- Development and implementation of a project for promoting early child and youth development in remote areas under the royal initiative of Her Royal Highness Princess Maha Chakri Sirindhorn Siam Boromrajakumari, Funds for Development of Children and Youth in Remote Areas, Department of Livestock Development.[18]	H	L	L	M	L
1.9.2 Establish regulations and standards for social support programmes based on national and international dietary guidelines.	Development and implementation of Blue Flag Project (Tong-Fa) by Department of Internal Trade.[19]	M	L	L	M	L
1.9.3 Incentivize local production of fruit and vegetables, such as urban agriculture.	Development and implementation of hospital-based food safety policy, by the Office of the Permanent Secretary of Ministry of Public Health. The hospitals will purchase fresh fruits and vegetables from local producers directly. [21]	M	M	L	M	L
2.1.1 Develop and implement evidence-based, targeted and appropriately funded, public education campaigns on the importance of physical activity.	- Development and implementation of Thai ChOPA & ChiPA Game Society" (physical activity innovation), initiated by Bureau of Physical Activity and Health, Department of Health.[22]	M	L	M	M	L

ECHO's specific policy action	Thailand's implementation	Assessment results by criteria				
		COM	COV	M&E	MSC	COH
	- Development and implementation of Play-by-age (bringing Thai children to change the world) Policy, by Bureau of Physical Activity and Health, Department of Health.[23]					
2.1.2 Update existing materials, as necessary, to include guidance on physical activity throughout the life course.	Development of guidelines for promoting physical activity, reducing sedentary behavior and promoting good sleep in each age group of Thai population, by Bureau of Physical Activity and Health, Department of Health.[24]	M	L	L	M	L
2.1.3 Disseminate guidance on physical activity to children, carers, school staff and health professionals in an accessible manner.	Development of guidelines for promoting physical activity, reducing sedentary behavior and promoting good sleep in each age group of Thai population, by Bureau of Physical Activity and Health, Department of Health.[24]	H	L	L	M	L
2.1.4 Use peer education and whole-of-school initiatives to influence the physical activity behaviors of children and social norms.	-	**Not available**				
2.2.1 Provide, in collaboration with other sectors (such as urban planning and transportation) and stakeholders, safe facilities, resources and opportunities for all children to be physically active during recreational time.	Office of the Permanent Secretary, Ministry of Education signed a memorandum of cooperation the Ministry of Tourism and Sports for opening a school area for public use to increase people's physical activity.[25]	H	L	L	H	L
3.1.1 Ensure that screening for hypertension and hyperglycaemia are included in antenatal care.	- Department of Health developed a Pink Book as an assessment tool for mothers and health professional to monitoring hypertension and hyperglycaemia in pregnant women.[3]<br>- Department of Health developed and provided medical examination guidelines for pregnant women for medical and public health personnel in health care facilities at each level – provincial, district to sub-district levels.[26]	H	H	M	M	M

ECHO's specific policy action	Thailand's implementation	Assessment results by criteria				
		COM	COV	M&E	MSC	COH
3.2.1 Ensure that measurement of weight and gestational weight gain are included in antenatal care.	Department of Health developed and provided medical examination guidelines for pregnant women for medical and public health personnel in health care facilities at each level – provincial, district to sub-district levels.[26]	H	H	M	M	M
3.3 Include an additional focus on appropriate nutrition in guidance and advice for both prospective mothers and fathers before conception and during pregnancy. (no specific action)	- Development and implementation of the Miracle of 1,000 Days (Maternal and Child Nutrition, Growth and Development) Policy, by Department of Health.[27]<br>- Department of Health in collaboration with UNICEF Thailand developed and implemented the Early Moment Matter on Mobile project to provide online information for parents and carers on early child development and care.[28]	H	H	M	H	M
3.4.1 Ensure that diet and nutrition counselling is included in antenatal care.	Department of Health developed a Pink Book as an assessment tool for mothers and health professional to monitoring and assess mother and child health.[3]	H	H	L	M	L
3.4.2 Include information on the association between prospective parents' diet, physical activity and health behaviors and the risk of childhood obesity in the curriculum of health care providers.	- Development and implementation of the Miracle of 1,000 Days (Maternal and Child Nutrition, Growth and Development) Policy, by Department of Health.[27]<br>- Department of Health in collaboration with UNICEF Thailand developed and implemented the Early Moment Matter on Mobile project to provide online information for parents and carers on early child development and care.[28]<br>- Development of a manual for intensive promotion on early childhood development in a hospital.[29]	H	H	M	M	L
3.4.3 Disseminate guidance and provide support for healthy diet and physical activity to prospective parents whom preconception or antenatal care may not reach.	Department of Health developed a Pink Book as an assessment tool for mothers and health professional to monitoring and assess mother and child health.[3]	H	H	L	M	L

ECHO's specific policy action	Thailand's implementation	Assessment results by criteria				
		COM	COV	M&E	MSC	COH
4.1 Enforce regulatory measures such as the International Code of Marketing of Breast-milk Substitutes and subsequent World Health Assembly resolutions. (no specific action)	Adoption of The 2017 Infant and Young Child Food Marketing Control Act in Thailand.[30]	H	H	M	H	M
4.2 Ensure all maternity facilities fully practice the Ten Steps to Successful Breastfeeding. (no specific action)	Development of Maternal and Child Health Standards with eleven steps to successful breastfeeding, and mandatory implementation of the standards in hospital setting. [31]<br><br>* Step 11 Not selling, distribute milk powder, milk bottles and fake nipples.	H+	H	M	M	L
4.3 Promote the benefits of breastfeeding for both mother and child through broad-based education to parents and the community at large. (no specific action)	- Department of Health developed a school manual for parents to serve as a guideline for school and health personnel in organizing activities for parents.[32]<br>- Department of Health developed a Pink Book as an assessment tool for mothers and health professional to monitoring and assess mother and child health. This book provides information for pregnant and postnatal women to take care of themselves and their children and for health personnel to advise mothers.[3]<br>- Department of Health in collaboration with UNICEF Thailand developed and implemented the Early Moment Matter on Mobile project to provide online information for parents and carers on early child development and care.[28]	H	L	M	M	L
4.4.1 Ratify ILO Convention 183 and enact legislation mandating all the provisions of ILO Recommendation 191 on maternity leave and provision of time and facilities in the work place for breastfeeding.	Department of Welfare and Department of Labor Protection, Ministry of Labor has revised the law, by extending a maternity leave from 90 days under the Labor Protection Act 2541, Section 41, to 98 days under the Labor Protection Act B.E. 2562, Section 7, which does not include antenatal leave or leave for pregnancy examination. The new Act is in line with International Labor Organization Convention No.183 on the right to protect maternity.[33]	H	H	M	H	H

ECHO's specific policy action	Thailand's implementation	Assessment results by criteria				
		COM	COV	M&E	MSC	COH
4.5.1 Assess the impact of legislation, regulations and guidelines to address the marketing of complementary foods for infants and young children, where required.	-	**Not available**				
4.5.2 Adopt and implement effective measures, such as legislation or regulation, to restrict the inappropriate marketing of complementary foods for infants and young children.	Enforcement of two laws: the Food and Drug Administration Act 2017 and the Food and Drug Administration announcement on regulations on food advertisement 2018, penalties being applied for both laws.[30]<br><br>Note: For food only, excluding beverages, and not evaluating nutrient content of a product.	M	H	M	H	H
4.5.3 Establish mechanisms to enforce effectively and monitor implementation of legislation or regulation on the marketing of complementary foods for infants and young children.	Enforcement of Food Supplement Marketing for Infants and Young Children. The government set up a committee to monitor and control the marketing of infant food and young children. The committee is responsible for recommending policies, strategies and measures to control the marketing of foods for infants and young child, in accordance with the Food Marketing Promotion Regulation for Infants and Young Children Act 2017 by the Ministry of Public Health.[30]	M	H	M	H	H

ECHO's specific policy action	Thailand's implementation	Assessment results by criteria				
		COM	COV	M&E	MSC	COH
4.6 Provide clear guidance and support to carers to avoid specific categories of foods (e.g. sugar-sweetened milks and fruit juices or energy-dense, nutrient-poor foods) for the prevention of excess weight gain. (no specific action)	Development of child caregiver training courses, operated by the Ministries of Health, Education, and Social Development and Human and Labor. The curriculum may vary, but the main content is taken from the Ministry of Public Health. There are also other materials.<br>• A child caregiver training manual for a child care center for nutrition surveillance, supported and monitored Bureau of Health Promotion, Department of Health.[29]<br>• A book on early childhood competency in children age 3-5 years, for guiding caregivers and school teachers, by the Office of Educational Standards and Learning Development. Ministry of Education.[34]<br>• Guidelines for early childhood development and care based on individual's competency in children age 0-5 years, by the Ministry of Education.[34]<br><br>It also includes guidance for caregivers to avoid providing certain foods.<br>• Development of infant feeding guidelines, by Dental Health Division together with Sweet Enough Network and Thai Health Promotion Foundation.[35]<br>• Development of a healthy snack for Thai children manual, by Healthy Kids Healthy Food Project and Bureau of Nutrition.[36]	H	H	L	M	L
4.7 Provide clear guidance and support to caregivers to encourage the consumption of a wide variety of healthy foods. (no specific action)	Development of child caregiver training courses, operated by the Ministries of Health, Education, and Social Development and Human and Labor. The curriculum may vary, but the main content is taken from the Ministry of Public Health. There are also other materials.<br>• A child caregiver training manual for a child care center for nutrition surveillance, supported and monitored Bureau of Health Promotion, Department of Health.[34]	H	H	L	M	L

ECHO's specific policy action	Thailand's implementation	Assessment results by criteria				
		COM	COV	M&E	MSC	COH
	• A book on early childhood competency in children age 3-5 years, for guiding caregivers and school teachers, by the Office of Educational Standards and Learning Development. Ministry of Education.[35]<br>• Guidelines for early childhood development and care based on individual's competency in children age 0-5 years, by the Ministry of Education.[34]					
4.8.1<br>(1) the introduction of appropriate complementary foods, avoiding the use of added sugar or sweeteners;<br>(2) responsive feeding to encourage infants and young children to eat a wide variety of healthy foods;<br>(3) which foods and beverages high in sugar, fat and salt should not be given to infants and young children; and<br>(4) appropriate portion sizes for children of different ages.	Development of child caregiver training courses, operated by the Ministries of Health, Education, and Social Development and Human and Labor. The curriculum may vary, but the main content is taken from the Ministry of Public Health. There are also other materials.<br>• A child caregiver training manual for a child care center for nutrition surveillance, supported and monitored Bureau of Health Promotion, Department of Health.[37]<br>• A book on early childhood competency in children age 3-5 years, for guiding caregivers and school teachers, by the Office of Educational Standards and Learning Development. Ministry of Education.[38]<br>• Guidelines for early childhood development and care based on individual's competency in children age 0-5 years, by the Ministry of Education.[34]<br><br>Other actions included development of recommendations for age-based supplementation guidelines for babies, by Bureau of Dental Health, and a healthy snack for Thai children manual, by Healthy Kids Healthy Food Project and Bureau of Nutrition.	H	H	L	M	L
4.8.2 Train community health workers or peer support groups to support appropriate complementary feeding.	Public Health Support Division, Department of Health Service Support, Ministry of Public Health provided trainings for health workers for a standard village health volunteer course and for	H	H	L	M	L

ECHO's specific policy action	Thailand's implementation	Assessment results by criteria				
		COM	COV	M&E	MSC	COH
	specific courses on maternal and child health, and reproductive health. [39]					
4.9.1 Set mandatory nutrition standards for foods and beverages provided (including meals) or sold (including vending machines and school shops) in public and private child-care settings or institutions.	- Development of a food, snacks, milk and drinks classification guide for children age 3-15 years (revised version) by Bureau of Nutrition.[14]<br>- Adoption of a National Early Childhood Development Center B.E. 2561, proposed by the Ministry of Education. [12]<br>- Development of Thai School Lunch standard.[13]	M	H	L	M	L
4.9.2 Implement such food laws, regulations and standards into catering services for existing child-care and other relevant settings.	- Development of a food, snacks, milk and drinks classification guide for children age 3-15 years (revised version) by Bureau of Nutrition.[14]<br>- Development of Thai School Lunch standard.[13]	M	H	L	M	L
4.10.1 Develop nutrition, food and health education curricula jointly between education and health sectors. Train teachers in curriculum delivery.	-	**Not available**				
4.10.2 Integrate nutrition and health education components, including practical skills, developed in collaboration with the education sector, into the core curriculum.	-	**Not available**				
4.11.1 Set standards for physical activity in child-care settings.	- Adoption of recommendations for promoting physical activity, reducing sedentary behaviors and sleep in each age group. To guide the guidelines for the general public (2017)[40]	H	N/A	L	M	L
4.11.2 Provide guidance to carers on the provision of safe and developmentally-appropriate physical activity, active play and active recreation for all children.	- Development of guidelines for promoting physical activity, reducing sedentary behavior and promoting good sleep in each age group of Thai population, by Bureau of Physical Activity and Health, Department of Health.[40]	H	N/A	L	M	L

ECHO's specific policy action	Thailand's implementation	Assessment results by criteria				
		COM	COV	M&E	MSC	COH
	- Development and implementation of Play-by-age (bringing Thai children to change the world) Policy, by Bureau of Physical Activity and Health, Department of Health.[23]					
4.12.1 Develop guidance on physical activity for children under 5 years of age, including age-appropriate activities and ideas to support and encourage participation in physical activity at home and in the community all year round.	Development of guidelines for promoting physical activity, reducing sedentary behavior and promoting good sleep in each age group of Thai population, by Bureau of Physical Activity and Health, Department of Health.[40]	H	N/A	L	M	L
4.12.2 Develop guidelines on appropriate sleep time and use of screen-based entertainment by children and adolescents (see recommendation 2.1) and ideas to avoid sedentary activities, including avoiding excessive screen-time, and to model regular physical activities for families.	Inclusion of recommendations on the appropriate sleep duration of children in each age group in a Pink Book (Part 3), Department of Health.<br><br>Other materials include:<br>- a parent manual for birth-6 years, by the Ministry of Social Development and Human Security,[41]<br>- recommendations for promoting physical activity, reducing sedentary behavior and promoting good sleep for early childhood age (birth-5 years), by the Bureau of Physical Activity for Health, Department of Health, and [42]<br>- recommendations for appropriate on-screen entertainment time in the Handbook of Promotion of Newborn Development from birth to 5 Years for carers and child care centers, by the Health Promotion Bureau, Department of Health.[43]	H	N/A	L	M	L
4.13.1 Conduct public awareness campaigns and disseminate information to increase awareness of the consequences of childhood obesity.	Development and implementation of the Miracle of 1,000 Days (Maternal and Child Nutrition, Growth and Development) Policy, by Department of Health.[27]	H	H	M	M	L

ECHO's specific policy action	Thailand's implementation	Assessment results by criteria				
		COM	COV	M&E	MSC	COH
4.13.2 Promote the benefits of physical activity for both carers and children through broadbased education to carers and the community at large.	Development and implementation of the Miracle of 1,000 Days (Maternal and Child Nutrition, Growth and Development) Policy, by Department of Health.[27]	H	H	M	M	L
4.13.3 Promote communication and community participation to raise awareness and create an enabling environment and social demand for policy action to improve diet and physical activity in children.	- Development and implementation of the Miracle of 1,000 Days (Maternal and Child Nutrition, Growth and Development) Policy, by Department of Health.[27]<br>- Adoption of the Child Center Standards and development of the standards manual, by Bureau of Health Promotion in collaboration with local community and relevant stakeholders.[37]<br><br>Other activities include implementation of the Pat Sports Playground Project, by the National Housing Authority, Bangkok Metropolitan Administration, Expressway Authority of Thailand, and many other related agencies.[44]	H	H	M	M	L
4.13.4 Identify community champions/leaders/civil society organizations to work with, and ensure community representation.	Development and implementation of the Miracle of 1,000 Days (Maternal and Child Nutrition, Growth and Development) Policy, by Department of Health.[27]	H	H	M	M	L
5.1 Establish standards for meals provided in schools, or foods and beverages sold in schools that meet healthy nutrition guidelines. (no specific action)	- Implementation of Thai School Lunch Software Program for preparing and providing school food meeting food standards.[13]<br>- Development of a school lunch management standard that was developed based on nutrition and food hygiene and safety standards for primary school in Thailand. [14, 45]<br>- Development of a food, snacks, milk and drinks classification guide for children age 3-15 years (revised version) by Bureau of Nutrition.[14]<br>- Development of a school lunch management guideline for single dish menu, developed by Nutrition Behavior Development Division, Bureau of Nutrition.[15]	H	H	M	M	L

ECHO's specific policy action	Thailand's implementation	Assessment results by criteria				
		COM	COV	M&E	MSC	COH
	- Development of recipes for school-aged children, by the Office of the Lunch Project Office of the Permanent Secretary for Education.[17]					
5.2.1 Set mandatory nutrition standards for foods and beverages provided (including meals) or sold (including vending machines and school shops) in the public and private school environment.	- Development of guidelines on the classification of lunches (savory food) and snacks and foods provided and sold. The guidelines are available in the food, snacks, milk and drinks classification guide for children age 3-15 years (revised version), by Bureau of Nutrition.[14]<br>- Development of Health Promotion School standards (2015 revised version), by Bureau of Health Promotion.[16]<br>- Implementation of Health Literacy School Policy, by Bureau of Health Promotion.[46]	M	H	M	M	L
5.2.2 Implement such food laws, regulations and standards into catering services for existing school and other relevant settings.	Cooperation with schools to ban sale of carbonated drinks in schools, by the Office of the Basic Education Commission, Ministry of Education.[47]	M	H	M	M	L
5.3.1 Ensure all school and sports facilities provide free access to safe drinking water.	Inclusion of an indicator on adequate drinking water in Health Promotion School standards, by Bureau of Health Promotion.[16]	M	M	L	M	L
5.4.1 Develop nutrition, food and health education curricula jointly between education and health sectors. Train teachers in curriculum delivery.	-	**Not available**				
5.4.2 Integrate nutrition and health education components, including practical skills, developed in collaboration with the education sector, into the core curriculum.	Office of Academic and Educational Standards, Ministry of Education established the Basic Education Core Curriculum BE 2551, Health Education and Physical Education learning subjects.[48]	M	H	M	M	L

ECHO’s specific policy action	Thailand's implementation	Assessment results by criteria				
		COM	COV	M&E	MSC	COH
5.5 Improve the nutrition literacy and skills of parents and carers. (no specific action)	Development of guidelines for organizing nutrition training for mentors and those involved. This is included in the manual for the organization to promote Thai children to reach their full potential, by Bureau of Nutrition, Department of Health.[49]	M	N/A	L	M	L
5.6.1 Work with schools and communities to deliver skills through community classes/groups.	Development and Implementation of ‘Reduce study time, Increase learning time’ Policy, by Department of Health.[50]	M	H	L	M	L
5.7.1 Set standards for quality physical education in the school curriculum.	Bureau of Nutrition, Department of Health developed guidelines for the prevention and control of obesity in schoolchildren.[48]	H	H	L	M	L
6.1.1 Implement a context-appropriate multicomponent weight management protocol that covers diet, physical activity and psychosocial support services tailored to children and families.	Bureau of Nutrition, Department of Health developed guidelines for the prevention and control of obesity in schoolchildren.[51]	M	H	L	M	L
6.1.2 Align services with existing clinical guidelines and clearly configure the roles of primary health care providers for effective multidisciplinary work.	-	**Not available**				
6.1.3 Educate and train concerned primary health care providers in identification and management of childhood obesity and associated stigmatization.	-	**Not available**				
6.1.4 Include childhood weight management services as part of universal health coverage.	-	**Not available**				

Abbreviation:
Criteria: COM – comprehensiveness, COV – coverage, M&E – monitoring & evaluation, MSC – multisectoral collaboration, COH – coherence

Assessment level: H – high, M – moderate, L – low

References

1. Daoduang P, Loysongkroa J. *Guidelines for the prevention of obesity in schoolchildren*. WVO officer of printing mill; 2014.
2. The Working group is to create a healthy diet for Thai people. *Guide to the flags of nutrition*. 3rd ed. WVO officer of printing mill; 1999.
3. Bureau of Health Promotion. *Mother and child health handbook*. The Veteran Press; 2018.
4. Bureau of Dental Health. World Milk Day Updated 31 May 2019. Accessed 1 March, 2019. https://multimedia.anamai.moph.go.th/infographics/world-milk-day/
5. Ministry of Finance. Excise Act B.E. 2560. In: Excise Department, editor. Bangkok: Ministry of Finance; 2017, 16 September. p. 111.
6. Food and Drug Administration. Healthier choice. Institute of Nutrition, Mahidol University. Accessed 1 March, 2019. http://healthierlogo.com/%E0%B8%82%E0%B9%89%E0%B8%AD%E0%B8%A1%E0%B8%B9%E0%B8%A5%E0%B9%82%E0%B8%84%E0%B8%A3%E0%B8%87%E0%B8%81%E0%B8%B2%E0%B8%A3/%E0%B8%84%E0%B8%A7%E0%B8%B2%E0%B8%A1%E0%B9%80%E0%B8%9B%E0%B9%87%E0%B8%99%E0%B8%A1%E0%B8%B2/
7. National Bureau of Agricultural Commodity and Food Standards. Codex Alimentarius Commission - CAC. National Bureau of Agricultural Commodity and Food Standards, Ministry of Agriculture and Cooperative. Accessed 1 March, 2020. https://www.acfs.go.th/codex/about.html
8. Ministry of Public Health. Notification of the Ministry of Public Health (No. 182) B.E. 2541 (1998) Re : Nutrition Labelling. In: Ministry of Public Health, editor. Nonthaburi: Ministry of Public Health; 1998,11 June.
9. Ministry of Public Health. Notification of the Ministry of Public Health (No. 219) B.E. 2544 (2001) Re : Nutrition Labelling (No. 2). In: Ministry of Public Health, editor. Nonthaburi: Ministry of Public Health,; 2001, 26 July.
10. Ministry of Public Health. Notification of Ministry of Public Health (No. 392) B.E.2561 (2018) Issued by virtue of the Food Act B.E. 2522 Re : Nutrition Labelling (No. 3). In: Ministry of Public Health, editor. Nonthaburi: Ministry of Public Health; 2018, 14 September.
11. Ministry of Public Health. Notification of Ministry of Public Health (No. 394) B.E.2561 (2018) Issued by virtue of the Food Act B.E. 2522 Re : Food products Required to bear Nutrition Labelling and Guideline Daily Amounts, GDA Labelling. In: Ministry of Public Health, editor. Nonthaburi: Ministry of Public Health; 2018, 26 September.
12. Office of the Education Council. *National standard for early childhood care, Development and education Thailand*. Prigwhan graphic co., ltd; 2019.
13. National Electronics and Computer Technology Center. Thai school lunch. Ministry of Higher Education, Science, Research and Innovation. Accessed 1 March, 2019. https://www.thaischoollunch.in.th/
14. Saereesuchart W, Srisura W, Thongbuasirilai N. *Food, Snack, Milk and beverage classification guide for nutritional standards for children aged 3-15 years (Revised version)*. WVO officer of printing mill; 2017.
15. Bureau of Nutrition. *Lunch management guidelines for "School Age Children", an alternative one dish menu*. Bureau of Nutrition.
16. Bureau of Health Promotion. *Health promoting school*. 3rd ed. Bureau of Academic Services; 2015.

17. Ministry of Education. *Recipes for school-aged children*. Express Transport Organization; 2004.
18. Royal Project Foundation. Children and youth development fund in the wilderness Royal Project Foundation. Accessed March 1, 2019. http://www.psproject.org/Funds.htm
19. Department of Internal Trade of Thailand. Thongfah. Ministry of Commerce. Accessed 1 March, 2019. https://thongfah.dit.go.th/AboutUI.aspx
20. Department of Children and Youth. Subsidy for the care of newborn children. Ministry of Social Development and Human Security,. Accessed 1 March, 2019. https://csg.dcy.go.th/
21. Department of Mental Health. *Food safety hospital*. 2nd ed. Born to be publishing co.,ltd.; 2017.
22. Department of Health. Thai ChOPA & ChiPA game society. Ministry of Public Health. Accessed 1 March, 2019. https://chopachipa.org/Home/content/news
23. Ministry of Public Health. Ministry of public health joins forces with 3 main organizations to promote a policy of inviting Thai children "Play to change the world". Ministry of Public Health. Updated 18 January 2020. Accessed 18 January, 2020. https://www.chiangmainews.co.th/page/archives/1236380
24. Khamput T, Puangkrampun M, Sangsamritpol W, Thongbo T, Seunglee S, Keryai T. *Recommendations for promoting physical activity reducing sedentary behavior And sleep for early childhood (newborn - 5 years)*. NC Concept company limited; 2017.
25. Office of the Basic Education Commission. OBEC opens a school sports ground encourage Thai people to exercise. Ministry of Education. Updated 28 November 2018. Accessed 1 March, 2019. https://www.obec.go.th/archives/27384
26. Institute of Medical Research and Technology Assessment. *Guidelines for necessary and appropriate health screening for pregnant women*. WVO officer of printing mill; 2016.
27. Bureau of Nutrition. *The miracle of 1,000 days: Maternal and child nutrition, growth and development*. Samchareon Phanich (Bangkok) co., ltd; 2018.
28. Department of Health. Early moments matter on mobile. Accessed 1 March, 2019. https://www.facebook.com/9YangTH/
29. Pattanapongthorn J, Thonghem A. *Early childhood development promotion manual for intensive hospital programs*. The Agricultural Co-operative Federation of Thailand; 2013.
30. Ministry of Public Health. Infant and Young Child Food Marketing Control Act B.E. 2560. Nonthaburi: Ministry of Public Health; 2017, 10 July.
31. Maternal and child health standards (Nonthaburi)
32. Department of Health. *Parents school guide*. Department of Health; 2015.
33. Ministry of Labor. Labor Protection Act No. 7 B.E. 2562. In: Department of Labor Protection and Wellfare, editor. Bangkok: Ministry of Labor; 2019, 4 April.
34. Office of the Education Council. *Guidelines for care and development of early childhood according to competencies to increase the quality of children according to the age of* 0-5. Prigwhan graphic co., ltd; 2018.
35. Bureau of Dental Health. *Guidance on complementary feeding for infant*. N-Mo printing and publishing limited partnership; 2007.
36. Vatanasuchart N, Chalaphirom P, Loungvara P, et al. *A guide to help Thai children to eat good desserts*. 4th ed. WVO officer of printing mill; 2017.
37. Bureau of Health Promotion. *A manual for the training of a child caregiver in a child center*. WVO officer of printing mill; 2006.
38. Bureau of Educational Standards and Learning Development. *Early childhood competencies in development with ages 3-5: guideline for child careers, teachers and teachers.* September 2009 ed. Plan for kids.co., ltd.; 2009.

39. Primary Health Care Division. *Village health volunteer standard training course (A.S.M.) Health managers by age group*. Ministry of Public Health; 2014.
40. Khamput T, Puangkrampun M, Sangsamritpol W, Thongbo T, Seunglee S, Keryai T. *Thailand recommendations on physical activity,*

Non-sedentary lifestyles, and sleeping. NC Concept Company Limited; 2017.
41. Department of Women's Affairs and Family Development. *Parenting guide (Newborn age - 6 years)*. Ministry of Social Development and Human Security; 2009.
42. Khamput T, Puangkrampun M, Sangsamritpol W, Thongbo T, Seunglee S, Keryai T. *Recommendations for promoting physical activity reducing sedentary behavior and sleep*

for early childhood (newborn - 5 years). NC Concept company limited; 2017.
43. Bureau of Health Promotion. *A guide to promoting the development of newborn babies - 5 years for caregivers. In family and child center*. WVO officer of printing mill.
44. National Housing Authority. Lan Kila Pat. National Housing Authority. Accessed 1 March, 2019. https://arsomsilparchitect.co.th/project/lankila-phat-1-kheha-khlong-chan-community/
45. Bureau of Nutrition. *Student lunch management guide according to nutritional standards food sanitation and food safety For elementary school.* Ministry of Public Health; 2015.
46. Health Center 8 Udon Thani. *Health literacy school : HLS*. Health Center 8 Udon Thani, Department of Health; 2018.
47. Request for cooperation to stop selling soft drinks in schools (Office of the Basic Education Commission, Ministry of Education) (2013, 11 October).
48. Ministry of Education. *The Basic Education Core Curriculum B.E.*2551 (*A.D.*2008). July 2008 ed. Printing House Agricultural Cooperatives of Thailand Limited; 2008.
49. Bureau of Nutrition. *Development guide to organization for promoting Thai children to grow to their full potential.* 3rd ed. WVO officer of printing mill; 2015.
50. Bureau of Academic Affairs and Education Standards. *Moderate class, More knowledge*. Bureau of Academic Affairs and Education Standards; 2015.
51. Bureau of Nutrition. *Smart kids coacher*. Samchareon Phanich (Bangkok) co., ltd.; 2016.
